# A Smart Monitoring System for Self-Nutrition Management in Pediatric Patients with Inherited Metabolic Disorders: Maple Syrup Urine Disease (MSUD)

**DOI:** 10.3390/healthcare11020178

**Published:** 2023-01-06

**Authors:** Haneen Reda Banjar

**Affiliations:** 1 Computer Science Department, Faculty of Computing and Information Technology, King Abdulaziz University, Jeddah 21589, Saudi Arabia; hrbanjar@kau.edu.sa; 2 Centre of Artificial Intelligence in Precision Medicines, King Abdulaziz University, Jeddah 21589, Saudi Arabia; 3 Center of Excellence in Genomic Medicine Research (CEGMR), King Abdulaziz University, Jeddah 21589, Saudi Arabia

**Keywords:** knowledge-based expert system, mobile application, food recognition, Maple Syrup Urine Disease

## Abstract

A metabolic disorder is due to a gene mutation that causes an enzyme deficiency which leads to metabolism problems. Maple Syrup Urine Disease (MSUD) is one of the most common and severe hereditary metabolic disorders in Saudi Arabia. Patients and families were burdened by complex and regular dietary therapy menus because of the lack of information on food labels, it was also difficult to keep track of MSUD’s typical diet. The prototype smart plate system proposed in this work may help patients with MSUD and their caregivers better manage the patients’ MSUD diet. The use of knowledge-based, food identification techniques and a device could provide a support tool for self-nutrition management in pediatric patients. The requirements of the system are specified by using questionaries. The design of the prototype is divided into two parts: software (mobile application) and hardware (3D model of the plate). The knowledge-based mobile application contains knowledge, databases, inference, food recognition, food plan, monitor food plan, and user interfaces. The hardware prototype is represented in a 3D model. All the patients agreed that a smart plate system connected to a mobile application could help to track and record their daily diet. A self-management application can help MSUD patients manage their diet in a way that is more pleasant, effortless, accurate, and intelligent than was previously possible with paper records. This could support dietetic professional practitioners and their patients to achieve sustainable results.

## 1. Introduction

Inborn Error Metabolic (IEM) includes Maple Syrup Urine Disease (MSUD), which is one of the most common and severe types of amino acid disorders. MSUD is defined by the inability to metabolize branched-chain amino acids (BCAAs), such as leucine (LEU), isoleucine (ILE), and valine (VAL), as well as their corresponding alpha ketone acids. High levels of the three amino acids in the blood and cerebrospinal fluid, α-ketoacids in urine, and development of the pathognomonic disease marker, alloisoleucine, all result from this deficiency, and the body’s organs, particularly the brain, suffer as a result [1]. MSUD care involves maintaining a strict lifelong diet, as well as taking the prescribed drugs and supplements, and closely monitoring blood tests [2]. Nutritional management leads to patients’ natural growth and development, or at the very least improves their condition and decreases the likelihood of severe problems in the future. The nutritional balance of the three amino acids between the required amount for growth and the harmful level for the patient should be carefully controlled. MSUD is a common disease in Saudi Arabia; as shown by the National Health and Nutrition Examination Survey (NHANES), the prevalence of metabolic syndrome was around 35% [3].

Many MSUD patients have not adequately monitored risk factors for the disease. One of the most challenging factors and necessary activities is self-management of nutrition, which poses a challenge for MSUD patients in general, and children and their families, owing to a knowledge gap between clinical recommendations and management in everyday practice. Patients and families are burdened by complex and regular dietary therapy menus. Secondly, it is also challenging to keep track of MSUD’s regular dietary intake since this detail is not often provided on food labels, putting patients and their caregivers at risk of overestimating or underestimating the amino acid content, leaving them feeling constrained and unable to eat a broad variety of foods. Both barriers endanger the possibility of successful treatment.

There are several methods for managing MSUD patients’ feeding but using conventional methods such as a paper-and-pen food diary or even digital ones that rely on manual tracking of each food intake and each protein consumed, as well as other essential nutritional details and portion sizes, is often a time-consuming, unreliable, and impractical process. However, several studies have investigated the use of smart device applications to enable users to effectively control their diet [4,5,6]. The application of computer vision by automatically defining nutrients, calculating nutrient values and serving size, and making them highly accurate based on food images can be beneficial to automate the process of monitoring the nutrition [7].

MSUD pediatric patients and their caregivers would be able to better manage their MSUD diet using the proposed prototype for a smart plate system. The design of the prototype is divided into two parts: software (mobile application) and hardware (3D model of the plate). The aim is to enable MSUD pediatric patients to self-manage their MSUD diet and improve adherence. The research objectives are: (i) identify the requirement and specification of the smart plate system, (ii) design the framework for the knowledge-based system which includes: knowledge base, databases, inference, food recognition, food plan, monitor food plan, (iii) design the user interfaces of the mobile application, and (iv) design the prototype for the smart plate to assist MSUD patients. The proposed prototype would advise the right amount that should be taken for helping to manage food intake or indicate if the patient already ate enough during the day with recommendations. This topic has significance because the designed intelligent system will support metabolic disorder patients of all ages to stay informed about healthcare conditions before the arrival of major healthcare risks. This project’s value to pediatric patients is that images of their meals can be uploaded directly to a mobile app, where a food recognition algorithm can determine an approximation of the meal’s amino acids. It will be compared to the patient’s regular diet in terms of weight and amino acids. Patients with MSUD can use a digital self-management tool that keeps track of their food easier, more convenient, more accurately, and more intelligent than was previously possible with paper records

It is important to present the pathophysiology of MSUD in the medical background to know the effect of three main amino acids that are related to the managing of food intake in MSUD patients. Then, the technical background includes food recognition and health monitoring systems, which are the suggested technologies, that may improve compliance with medical nutrition treatment and metabolic management by improving the precision of food tracking and information sharing between patients and healthcare professionals.

### 1.1. Medical Background

Decreased activity of the branched ketoacid dehydrogenase (BCKAD) enzyme complex leads to the metabolic disease MSUD. The enzymes, being essential to life, are proteins in their most basic form. Proteins are essential for human survival and development and can also be broken down and used as a source of quick energy. Amino acids come in 20 different forms and can be combined in a wide variety of functional ways to form proteins [8]. These proteins have a three-dimensional structure, the functional form of all proteins that facilitate interactions with other proteins, and thus the performance of a wide range of biological tasks. A protein’s or an enzyme’s biological activity can be altered or negated by even the simplest alteration in the protein’s or enzyme’s structure. Changes in expression or protein composition attributable to mutations in these genes have been linked to a variety of diseases [8].

The three branched-chain amino acids (BCAAs) (isoleucine, leucine, and valine) are essential nutrients [1]. To be used by the body, amino acids must be processed by specific enzymes. Reverse transport is the initial stage in BCAAs catabolism, and it involves a transaminase and pyridoxal phosphate enzyme [1]. This step involves the synthesis of alpha-keto acids from leucine, isoleucine, and valine [2]. Pathogenic deficiencies in these components lead to elevated BCAA levels in the body, resulting in MSUD and leading to a variety of symptoms such as brain damage, skeletal muscle and immune system dysfunction, coma, and possibly fatal diseases [2]. Patients with MSUD have a wide range of neurological issues due to an anomaly in the synthesis of glutamate, a neurotransmitter in the central nervous system. This abnormality results from thiamine pyrophosphate saturation of its location on the BCKAD [1]. Severe neurological and other organ damage can be avoided with prompt and accurate therapy. It might be difficult for both the patient and their family to observe their cultural and social routines while adhering to a metabolic diet. Thus, food recognition technology could assist in managing food intake.

### 1.2. Technical Background

#### 1.2.1. Food Recognition

Many diseases can be managed by paying more attention to one’s nutrition, and numerous studies have looked into the usefulness of mobile applications for this purpose [9]. Convolution Neural Networks (CNN) and other network architectures used in deep learning have been tested in the field of food image recognition and volume estimation [10]. CNNs can extract features at multiple levels and may override human performance in some applications (such as face recognition) [11]. So far, researchers have developed computer vision-specific pipelines for either food items (such as meals) or retail packaging. For example, FoodNet101 contains 101,000 images of labeled dishes [12] while SKU110 contains 1.74 million images of retail products with a shelf life of packages [11]. The publication of image data sets is critical to the advancement of resume solutions. The effectiveness of deep learning techniques is highly dependent on the size of the available data set for training. With the success and great improvement of CNN-based methods, it has been employed in many smartphone applications [10]. The studies focus on food classification, cross-modal retrieval, ingredient analysis, and volume estimation [11].

Food classification is considered in two areas: weight estimation and calorie estimation. Firstly, in the weight estimation, He et al. [13] created a method of monitoring one’s diet by comparing photographs of food consumed throughout each meal. The photos of the meal are then examined to extract the item’s nutrient content using a form template for foods with regular shapes and area-based weight estimation for foods with irregular shapes. Ege et al. [10] examined three prior research and proposed two creative solutions to determining food portion sizes. The first one is “CalorieCam” which is a food calorie estimation system by using a reference object on an Android smartphone. The item serves as a reference object, and it may take the form of anything as commonplace as a wallet or a card somewhat larger than a credit card. The second one is a weakly-supervised segmentation-based food calorie estimation. Multiple meals’ calorie counts can be extracted from a single image. The third one is “AR DeepCalorieCam V2”, Based on the optical visual-inertial iodometry measurement option in iOS ARKit, this system was developed for iOS devices to provide rough estimates of the volume and calorie content of real food. Authors in [10] decided to take advantage of the close relationship between calorie count and food type and proposed a system that would calculate calorie count straight from recipe site contents and preparation instructions. To do this, they created a database of food photos annotated with calorie counts based on the components stated on the recipe cards. When a user enters a new food item, the system compares it to the photos of foods already in the database to determine how many calories it is likely to contain. To automatically estimate calories from an image, the theories assert that simultaneous training of the model on the food recognition and calorie estimation tasks may increase the performance of algorithms more than standalone training.

#### 1.2.2. Healthcare Monitoring Systems

The technological applications in healthcare are supporting patients to maintain a healthy lifestyle after proper adjustment in daily life habits and behaviors. Mougiakakou et al. [14] designed a platform for type 1 diabetic patients. This platform helped in the monitoring, management, diagnosis, and treatment of the disease. The researchers used database and data mining techniques along with simulation algorithms. The device has a connection with the laptop to transfer data and communicate about the condition of patients with a healthcare professional. Similarly, Piniewski et al. [15] worked on a technological application that can help patients better monitor their healthcare disorders. Moreover, Hommersom et al. [16] worked on MoSHCA for implementing a technological application for improving the interaction between patients and doctors along with supporting patients to manage chronic diseases. It is very impactful as the number of patients with chronic illness is increasing day by day all over the world. MoSHCA is a user-friendly, intelligent, and secure device which can support decision-making practices by using sensors for monitoring and embedded software for data transferring.

### 1.3. Related Applications

Regarding the related applications, there is only one publication associated with website applications for managing the diet of patients with IEMs called the Metabolic Diet App [17]. In addition, DietAssistant For MSUD [18] was developed to get the Leucine content of patients’ consumed products. However, a few works, in mobile app stores, supported Phenylketonuria (PKU) as one type of IEM. Table 1 summarized applications that had been reviewed. The table presented smartphone applications for IEMs such as Metabolic Balancer [19], PKU calculator [20], AccuGo for PKU [21], My Diet For PKU [22], and PKU Diet [23]. In summary, all the applications did not use artificial intelligence and computer vision to detect the food automatedly from the images

## 2. Materials and Methods

To create a high-quality product, the SDLC (Software Development Life Cycle) outlines the phases of software development: requirements and specification, system design, initial implementation (prototype), and testing. The hardware is simulated using a 3D figure as an initial design before implementation.

### 2.1. Requirements and Specifications

Qualitative research approaches rely on acquiring data through conversational communication. These strategies are designed to assist in exposing the behaviors and opinions of a group on specific subjects. The most common tool employed in the qualitative research method is the questionnaire. The requirements and specifications are identified by using a questionnaire. The following questions and the possible answers were implemented in the google form. The questionnaire is sent to MSUD patients, and they sign the consent form by agreeing at the end of the form to submit their answers and information. Table 2 shows the questions and possible answers.

### 2.2. System Design

**Figure 1:**
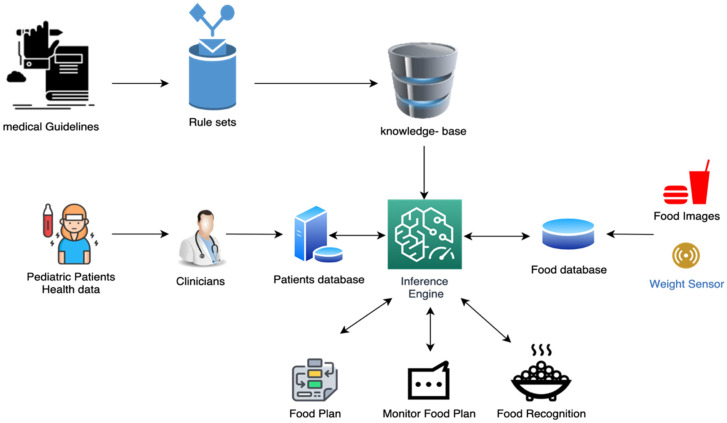
Framework for the system.

The design of the prototype is divided into two parts: software (mobile application) and hardware (3D model of the plate). The software included three subsystems: (i) knowledge-based expert system (knowledge acquisition from medical guidelines, knowledge base, inference engine, and user interface), (ii) information management system (electronic health record, food plan, and monitor food plan), and (iii) food recognition model. The second part is the hardware which includes a 3D model for a smart plate. The plate assists in receiving the input for the food recognition model (image and weight). In Figure 1, the system components are demonstrated and the details for each subsystem are explained.

### 2.3. Knowledge-Based System (Software)

#### 2.3.1. Knowledge Acquisition from Medical Guidelines

Knowledge acquisition is the process of gaining information from various sources, such as humans with expertise, official documents such as books and guidelines, or electronic databases [24]. To begin, knowledge engineers look for information about MSUD and explore the medical area in greater depth. A review of medical guidelines was employed in this study to collect information about the food’s amino acids. To get the knowledge, the guideline in [1] is used. To establish a prescription for long-term nutrition support, it is recommended in [1] to consider: patient age, growth rate, adequacy of energy and protein intake, and state of health. The main plan includes ILE, LEU, VAL, protein, energy, and fluid. The lowest values for (ILE, LEU, and VAL) are suggested in the patient food plan in Table 3. The ruleset contains the rules from the clinician who supervises the patient status, and it is in If and Then form. The age, ILE, LEU, VAL, protein, energy, and fluid are calculated after each meal on a daily basis, and the food intake should not exceed the food plan that is prescribed by the clinician.

#### 2.3.2. Knowledge Representation

Knowledge representation is the process of organizing acquired knowledge so that it can be applied to use. The steps in this process are encoding the information in the knowledge base and creating a knowledge frame as tables [24]. The knowledge shows how the clinician maps the food intake and food weight estimation to the prescribed food plan using a published nutrient content of high- and low-protein foods at the 100-g serving size; see Table S2 (Supplementary Material).

#### 2.3.3. Knowledge Verification and Validation

For knowledge to be of sufficient quality, it must be subjected to a process of validation and verification. In order to ensure correctness, knowledge is usually presented to a domain expert or experts [24]. In this project, a pediatric clinician who is specialized in metabolic disease from King Abdelaziz hospital provided the researcher with the medical guidelines and verified the steps of creating the knowledge base.

#### 2.3.4. Inference Engine

Programming inference logic into a computer so it may apply previously acquired knowledge to a new situation is at the basis of this project. The technology can then give recommendations to individuals who are not experts in field [24]. The inference code is written in python.

#### 2.3.5. User Interface

There are two users who can use the mobile application: patients and clinicians. The functionality of the application allows both users to sign up and log in to the app. Firstly, clinicians can view a list of patients, add new patients, or delete any patients, select the patient from the list and view his/her clinical summary (physical data, laboratory data, and nutrient intake data), chat with the patient, edit the physical data after each patient visit. Secondly, the patient can display previous options (physical, laboratory, and food intake data), his/her food management plan, food database, food monitoring, and chat with experts. Manually entering food consumption information or taking a picture with a camera are both options for patients and clinicians. When a user’s diet intake approaches or surpasses the prescribed limits, the app notifies them and suggests they choose a different item from the database.

### 2.4. Information Management System

The information management system includes Electronic Health Records (EHR), food plans, and monitored food plans. The EHR store physical, laboratory, and food intake data. Firstly, the patient’s nutrition status can be estimated based on the physical signs noticed during the clinical examination. Young children are a more obvious and valuable population for physical examination. However, early physical symptoms of sickness are not usually present. The results of a physical examination might give a rough idea of the patient’s nutritional status, but other methods are typically used to fill in the details. Secondly, the samples for laboratory tests are often taken from patients at medical centers, and the patients’ dietary assessments can benefit greatly from laboratory results. Clinicians order those tests for diagnosis. Finally, food intake data assist in identifying those who may need nutritional care and indicate where to upgrade nutrition support services. In addition, more options are added for nutrition intervention programs and procedures for evaluation and monitoring are planned. The creation of group profiles to use in identifying populations at nutritional risk and formulating intervention strategies is the primary goal of data collecting. For this reason, it is essential to collect data in a systematic manner that describes the nutrition issues facing the population being helped. The key to the profile’s successful application may lie in tailoring it to the specific requirements of a certain healthcare organization.

#### Databases

The database has three tables: patient profiles as EHR explained in the previous section (information management system) and food tables. The food table includes food ID, food name, ILE, LEU, VAL, protein, energy, and fat. If the user selects the food from the list, the system uses the food ID to retrieve ILE, LEU, VAL, protein, and energy from the knowledge-base sample Table S2 (Supplementary Material). If the user uploads the image, the trained model will predict the food whether it is single or mixed food. The food approximation measures such as calories, weight, volume, and mass are related to the food image. In this research, the experiment of the image classification is on a single food or mixed food while the weight is sent by the hardware through Bluetooth to the application from the plate after connecting it with the application. To determine how many calories are in each image, the system estimates the volume of each serving and then retrieves the food components from tables. Then, calories were retrieved automatically from the database (Table S2 Supplementary Material) by calculating approximately 100 g serving size and estimated quantity to calculate the food contents.

### 2.5. Food Recognition

#### 2.5.1. Dataset

For a range of applications in eating habits and dietary assessment, food detection, classification, and analysis have been studied extensively. The research community needs a dataset of photos for testing and training on the specific topic of food classification of either single or mixed food items. The FooDD dataset [25] includes 3000 photographs captured with a range of cameras.

#### 2.5.2. Food Recognition Modeling

The method is implemented as described in [26,27,28]. Through this setup, a user’s mobile device will be able to capture images of their meal. Each image should have pre-processing and segmentation. At the pre-processing step, it is necessary to conduct a basic transformation on the image to shift the image size into a standard format for reliable segmentation results. It is possible that the image will be cropped or padded to make it fit into one of the predetermined size categories if its actual size is outside of the range. At the segmentation step, each image is analyzed by using color segmentations (k-mean clustering, and texture segmentation [28]). The k-mean method automatically determines the mean of a group of clusters when a stable distribution of cluster samples is reached. The techniques used for segmentation typically use the Euclidean distance to establish the degree of spatial or color similarity between adjacent pixels. Uniform intensity regions are the most common type of region model used for grayscale images. Clustering methods used for color image processing often work in extremely high-dimensional regions. The problem of area segmentation in color images is not as clearly defined as in grayscale images due to the additional complexity introduced by the necessity for three variables to represent color pixels. To obtain more accurate results during segmentation, the texture segmentation is added to the method. Gabor filter is used to estimate texture properties. Spatial coordinates of the pixels were included as features. Each of these Gabor filters produces an array of two dimensions that is exactly the same size as the original picture. The matching orientation and spatial frequency of the input picture is represented by the sum of all elements in such an array.

Training and testing data, both of which consist of certain data, are common requirements in classification. The most popular supervised learning technique in medical applications with images was the support vector machine (SVM) [26,27,28,29]. Therefore, the food recognition model is developed by SVM and OpenCV. Single or mixed food are the two possible categories from the model: apples, oranges, and slices of bread are examples of single foods, while plates of mixed foods such as salad, pizza, and kebabs with rice are examples of mixed foods. There is a single class label and many features associated with each image in the training set. The radial basis function (RBF) kernel is used to map samples into a higher dimensional space. Each food image had 18 features extracted during the segmentation phase and are used as the training vectors of SVM—five for texture, five for color, five for size, and three for shape. The model should predict the target food which is given by its features. After the food has been detected and the app has made a suggestion as to what it is, it is up to the user to either accept the app’s suggestion or make a correction in the mobile device.

#### 2.5.3. Validation and Evaluation of the Food Recognition Model

The classification model must have the ability to generalize. Here, the 10-fold cross-validation is used to minimize the bias associated with random sampling in training and testing data. The classification model is trained using all the samples except a single sample, which will hold for testing. The performance of the classification model is measured by using a confusion matrix. Here, the problem is a two classification where the first class represents a single food portion, and the other class is mixed food on a plate. In the single food evaluation, recognition rate using all features (10-fold cross validation) is calculated for each food item. In the mixed food evaluation, the dataset includes two types of mixed food: mixed food such as salad and non-mixed food such as two apples with an orange on a plate. The accuracy results are reported for mixed food and non-mixed food.

### 2.6. Part 2: Hardware Simulation

#### D Model Simulation for Smart Plate System

AutoCAD is used to draw the simulation of a 3D model and Arduino Integrated Development Environment (IDE) will be used to write the code for sending food images, and weight as well as connect to the application. The project also needs hardware materials such as a plate and the following main components in addition to a breadboard and some connecting wires: Arduino UNO, and Bluetooth. To begin using the Android app, you must first connect the Bluetooth module to your mobile device. In addition, the load cell (40 kg), HX711 Load cell amplifier module, 16 × 2 LCD, USB cable, and nut bolts are needed. Arduino Code is demonstrated in [30].

### 2.7. Implementation

In this project, Python is used for food recognition modeling, Proto.io will be used for designing the mobile application prototype, and Flutter will be used for the implementation of the mobile application. It has built-in Bluetooth to connect the plate to the mobile application. The scaler is for calculating the weight of the food and displaying it on the LCD screen. The weight also is sent to the mobile application. The camera will take the picture upon user request. The image will be sent automatically to the mobile application and the food recognition model will classify the image into one of the food categories in the food database. Based on the weight and amino acids, the prescription food plan for the patients will be compared with the daily food intake. Then, the details of the amino acid will be stored in the patient profile. If the amount exceeds the limit of the amino acid, the warning message will be displayed on the mobile application.

### 2.8. Testing

Distributing the prototype to a wide variety of users and collecting their input is an integral aspect of the prototyping process. In this study, the results of the food recognition model are presented as the 3D model of the device. The mobile application prototype is designed and feedback from the two clinicians is considered during the design.

## 3. Results

The SDLC phases are used to develop the system. All requirements and specifications were defined after collecting and analyzing the questionnaire responses. The results were divided into two parts: software (mobile application) and hardware (3D model of the plate). The results of the subsystems—knowledge-based expert system, information management system, and food recognition model—are demonstrated. The prototype of the second part included a 3D model for a smart plate.

### 3.1. Questionnaire Results

Of their 18 participants’ answers, 58 % were less than 12 years old while 35% were 12–18 years old. Most participants were MSUD-diagnosed by birth (82.4%). The parents were usually responsible for monitoring the food intake of 88.2% of participants. Among the participants, 17% had experienced a change of the food plan three times within a year, while 29.4% had to change the food plan twice and 53% had changed it at least once. A total of 53% of participants agreed that they had difficulty monitoring their food intake and staying within the limit of the prescribed plan. All the MSUD participants agreed on the answers of finding it helpful for a mobile application to give them a warning about what they are consuming. Only 11% found the manual method is better to monitor food intake while 88% found the mobile applications would be better than the manual method. Finally, 94% agreed to buy a smart device that helped with monitoring their food intake.

### 3.2. Knowledge-Based Expert System

#### 3.2.1. Knowledge Base

The amino acids included in the diet were determined by a review of medical recommendations for this investigation. Knowledge is obtained by following the instructions in [1]. The sample of the selected requirements for laboratory tests was shown in Supplementary Material Table S1. Based on the reported nutritional content of high- and low-protein meals at the 100-g serving size (see Supplementary Material Table S2), the information demonstrates how the physician maps the food intake and food weight estimation to the prescribed diet plan. After that, the expert in pediatric metabolic disorder from King Abdelaziz Hospital verified the use of the medical guidelines and double-checked the process of knowledge base construction.

#### 3.2.2. User Interface

Users are divided into two types: patients and clinicians. Both users can sign up and log in to the app. Clinicians display a list of patients who are registered in his/her clinic. Clinicians can add new patients or delete any patients. They can also select the patient from the list, view his/her clinical summary, and chat with the patient. The clinical summary includes physical data, laboratory data, and nutrient intake data. The clinician can edit the physical data after each patient visit. Laboratory data includes calcium, serum (Mmol/L), urine (Mmol/mg Cr), serum Phos (Mmol/L), serum 1,25-OH. D (pg/mL), Alk Phos (M/L), Ferritin (ng/mL), Hgb (g/dL), Albumin (/dL), platelets (×10/L), and Neutrophils (×10/L). Based on the laboratory data and physical data, the clinician adds the food plan for the patients.

**Figure 2:**
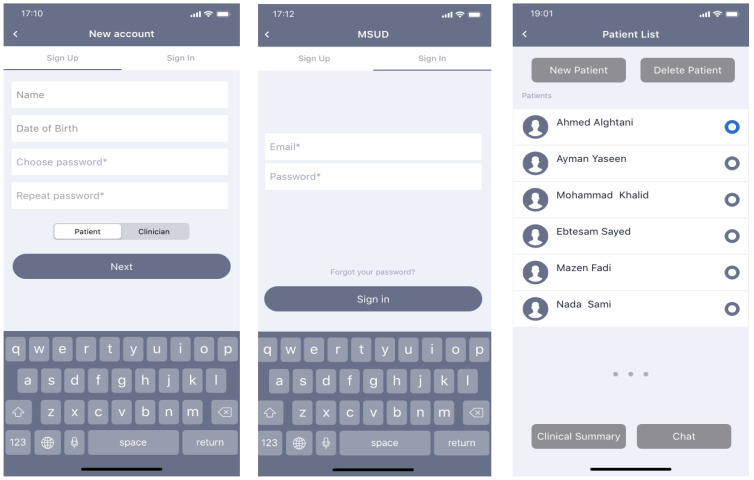

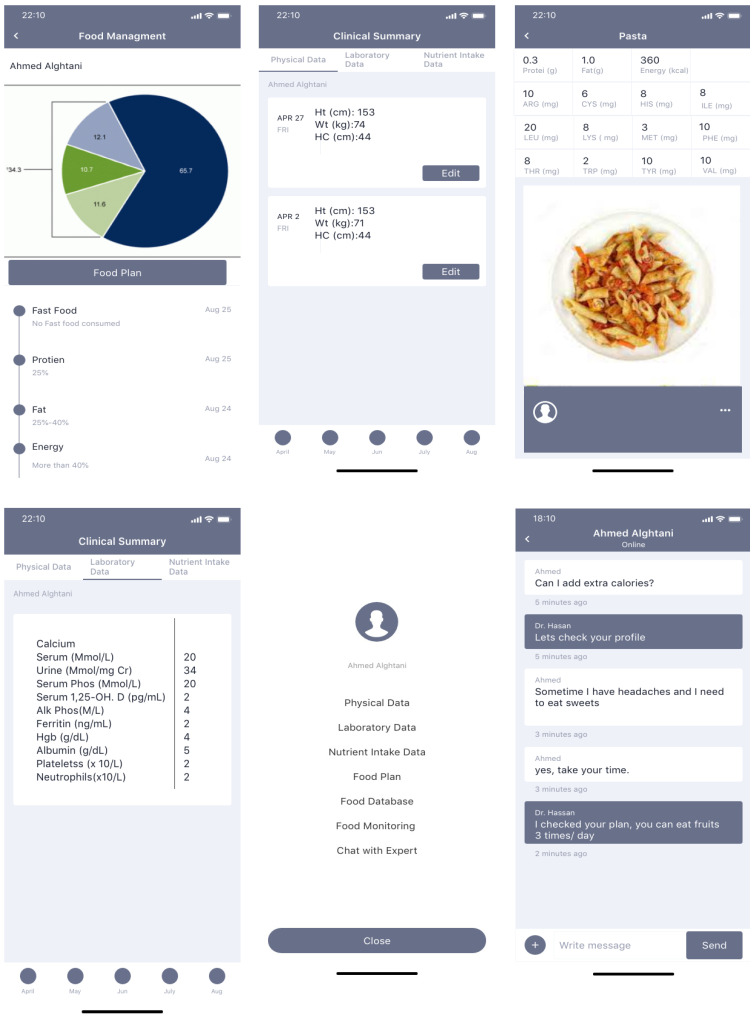

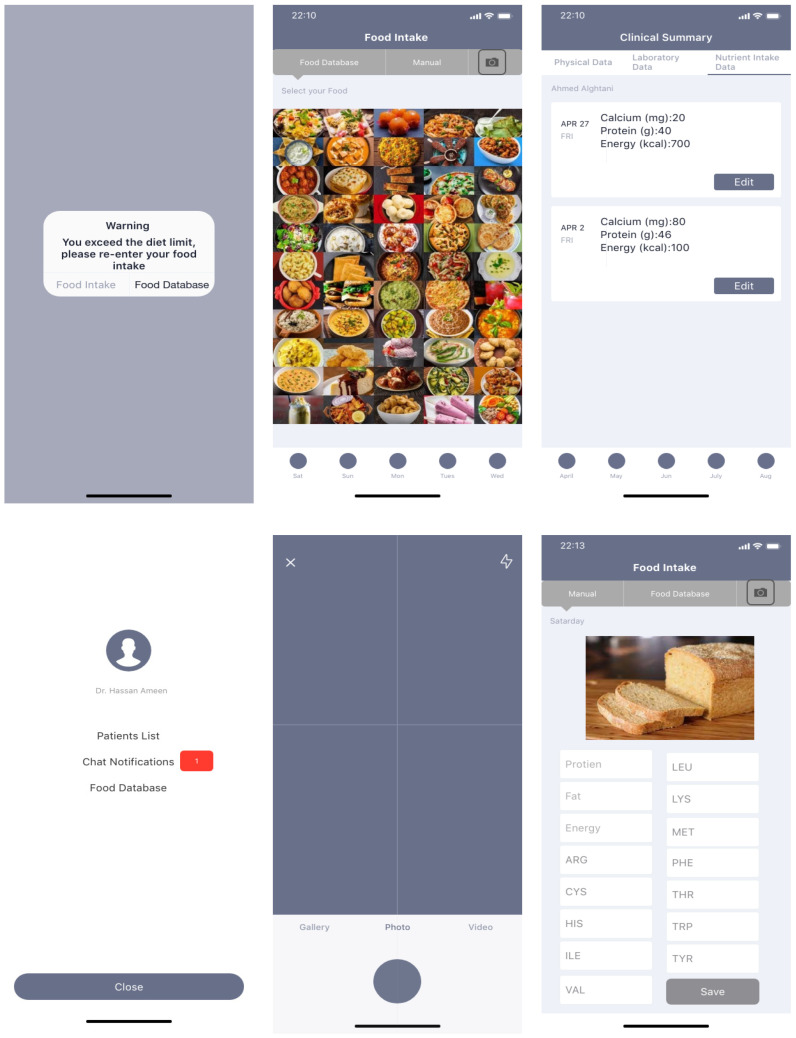
User interface for the mobile application to support MSUD patients and clinicians.

Each patient can display previous options (physical, laboratory, and food intake data) as well as his/her food management plan, food database, food monitoring, and chat with experts. The patient and clinicians can select food and display its details such as protein (g), fat (g), energy (kcal), ARG (mg), CYS (mg), HIS (mg), ILE (mg), LEU (mg), LYS (mg), MET (mg), PHE (mg), THR (mg), TRP (mg), TYR (mg), and VAL (mg). The patient and clinicians also can add a photo of the food using a camera and can also add food intake details manually. A comprehensive discussion that includes details on specific image processing and machine learning algorithms and design has been demonstrated in [25]. The weight of food is determined with the help of a smart plate camera device that can connect by Bluetooth. The system will allow food to be photographed and analyzed on a mobile device, and a response will be given to the user immediately. Patients receive warning messages if they exceed the diet limits, and the app recommends that patients select another option from the food database. All interfaces were demonstrated in Figure 2.

### 3.3. Information Management System

**Figure 3:**
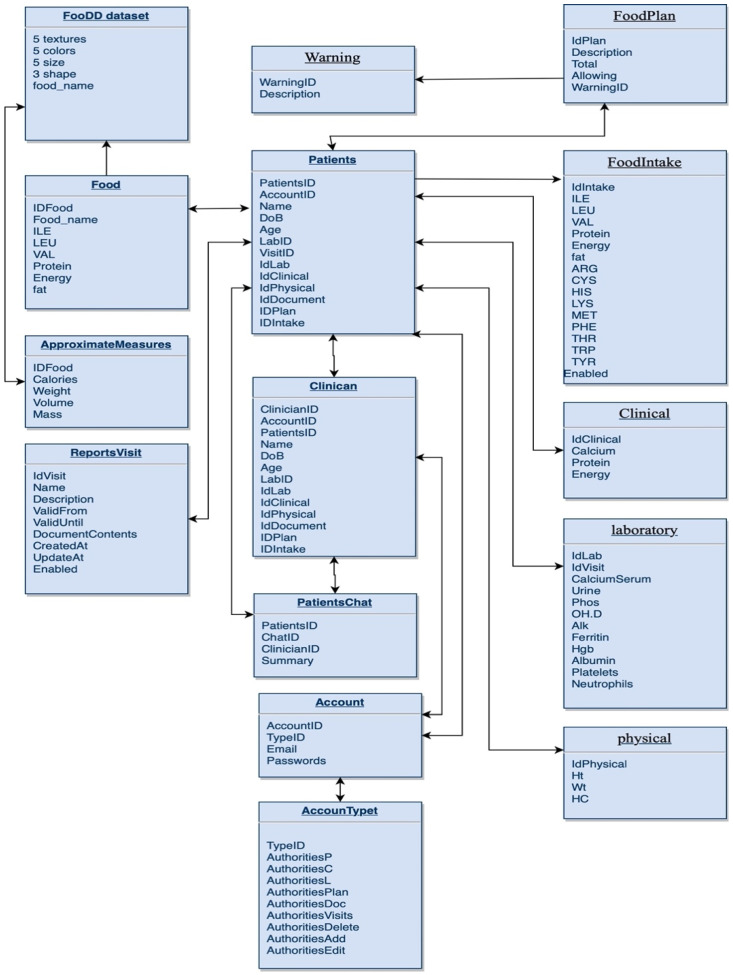
MSUD Database Schema.

The database included tables that were shown in Figure 3. HER for patients included details of his/her personal account, food plan, food intake, clinical, and physical data, and laboratory tests. Clinicians also shared some tables such as account and patient details. The database also included the images and knowledge about the food images and components.

### 3.4. Food Recognition Model Results

The results were divided into two experiments: single food and mixed food. From single food including fruits and a single piece of food, the training results ranged from 97–77%, and the total average was 91.30% (10-fold cross-validation) while the testing accuracy ranged from 92–85% and the total average was 89.3%. In the second experiment, there were around 3000 non-mixed plates and 500 mixed plates recorded in the database. In non-mixed food, three sets of images are trained each including 1000, 2000, and 3000. For each group, 1000 images were held for testing purposes. The training accuracy achieved with non-mixed food using SVM with 1000 was 80%, while it was 81% with 2000, and achieved the highest accuracy at 84% with 3000. In mixed food, 500 images are used and achieved training accuracy of 58%. The testing performance in the three groups were about 2–5% less than the training accuracy.

### 3.5. The 3D Modelling

**Figure 4:**
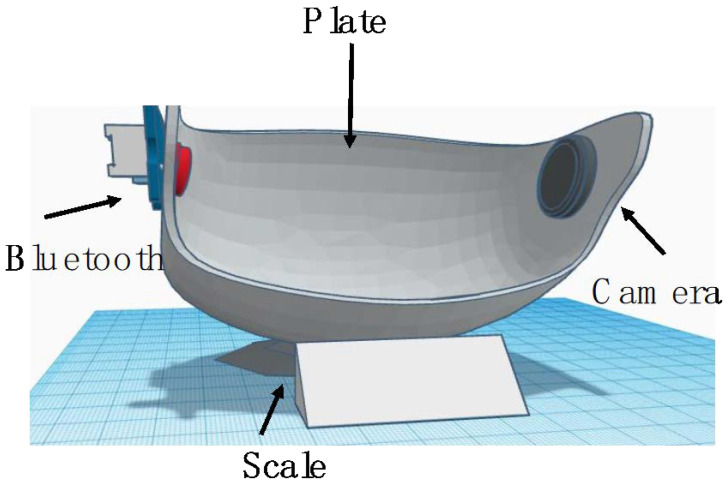
The 3D model for the plate.

Figure 4 shows a simulation design for the smart plate to be used by MSUD pediatric patients. Pediatric patients use the plate by putting the single food or mixed food. Then, the user should turn on the Arduino board, activate Bluetooth on the Android, then look for nearby devices to connect to take the food images or uploaded to the database in the application. The system retrieves all the food details after classification and computes the allowing quantity. The system notifies the user with the results, either a message to eat the food or warning message.

## 4. Discussion

With the rapid development of smartphone technology, people have grown to rely on them for a wide variety of tasks beyond merely making phone calls [9,31]. Keeping a food diary, taking pictures of meals, tracking fitness progress and calorie intake, and receiving encouraging feedback and advice on how to incorporate these things into one’s diet and daily routine are all made easier by the variety of applications available for smartphones [9]. Ho et al. [32] reported that people are more likely to use an app on their mobile device than on their computer. In addition to providing easy access, they also allow patients to take photos of their food with little to no effort. When patients do not have a connection to the internet, they can still use some of the features.

Dietary therapy for a wide variety of illnesses has incorporated the use of mobile health applications, which have been shown to improve self-monitoring and adherence [31,32]. Successful health app implementations include one for diabetes education, which allows patients to log food consumed, monitor blood sugar levels, communicate this information with their doctors, and set up text message reminders for when they need to take their medication. These apps have been found to be somewhat helpful for facilitating patient self-management. In addition, it increased the consumption of healthy foods such as fruits and vegetables [31].

Unfortunately, few programs take advantage of the benefits of a computer vision framework. Patients and dietitians both stand to gain from the incorporation of computer vision technology into such programs. Patients can use computer vision to automatically identify nutrients, calculate nutrient values, and estimate serving sizes from food photographs.

A digital self-management application can help MSUD patients manage their diet in a way that is more pleasant, effortless, accurate, and intelligent than was previously possible with paper records due to the proliferation of smartphones and the incorporation of cutting-edge technologies in artificial intelligence and computer vision.

MSUD treatment relied heavily on a strict lifelong diet along with taking necessary medications and supplements (according to the patient’s state of health) and closely monitoring blood tests. Since MSUD is considered degenerative, especially when nutritional control is lost, its bad symptoms appear from apnea, spasms, and others. These relapse symptoms usually range from bad to worst and some of them can be critical, even fatal. Conversely, good management of nutrition contributes to the normal growth and development of patients, or at least improves the patient’s condition and reduces the risk of serious complications in the future. Attention should be paid to the nutritional balance of the three amino acids between the important amount for growth and the harmful level for the patient. Additionally, catabolism must be prevented, and urine should be kept free of branched-chain keto acids. In this paper, we proposed a mobile expert system with computer vision technology as a support tool for MSUD patients (2–12 years) and their caregivers, to enable them to self-manage their MSUD diet and improve adherence to it.

The application ensures a healthy balanced diet for patients with synchronized food energy balance, tracks patients’ nutritional intake, and monitors protein, branched-chain amino acids (BCAAs) (Leucine (LEU), isoleucine (ILU), valine (VAL)) and fluid concentrations. Moreover, it reduces the risk of human error because the values of protein, amino acids, other nutrients, and portion size can be calculated automatically from a food image, beside the ability to manually select food menus from the application database. The amount of food with the calculated nutritional energy (calories, carbohydrates, protein, fat) and the three amino acids is given with each food item, and if any of them exceed the calculated marginal level, the user will get a notification if he/she has already taken enough quantity during the day with a recommendation. In addition, the application worked on planning a prescribed diet and suggesting various meals, which are made based on the information from the MSUD Patients’ guidelines [1]. In addition to providing a list of nutritional alternatives and providing users with feedback on nutritional information accompanied by insightful nutritional advice to make healthier lifestyle choices. It will have data on safe foods for patient. Thus, people with MSUD will be able to manage their diet more easily and accurately and will be able to share with their families eating a larger variety of foods without doubt about the correctness of their nutrient values, and they will be able to direct their time to something other than the lengthy search for appropriate meals. The result of monitoring the various analyses gave the nutrition expert sufficient and detailed data to verify the effect and quality of the diet followed, and it will motivate the patient and the parents to adhere. The following are the advantages from using the proposed application. Firstly, helping the dietitian and the patient’s caregivers to better understand the patient’s condition through dietary analysis based on blood tests. Secondly, fulfilling users’ desire and interest in monitoring what they eat, as it provides potential health benefits. Thirdly, helping children with MSUD lead an active and normal life through regular monitoring and careful attention to dietary restrictions, as the child’s condition can be improved, and possible complications can be avoided. Fourthly, helping alleviate the daily challenge of tracking the metabolic diet and meal planning for individuals living with metabolic nutrition therapies restricted by protein, amino acids, or organic. Finally, reducing patient relapses, thus reducing healthcare costs, and reducing mortality rates. There are some limitations such as estimating portion sizes and applications that require too many steps for data recording and frequent alerts that were a nuisance to deal with are only examples of the broader challenges faced by patients and dietitians [9].

The system could help patients to be aware of the internal condition of the body by measuring and comparing the values of harmful foods. They were also able to keep their family and healthcare providers with their current health situation. The mobile application could provide help to utilize these specified outcomes. The patients’ profiles with their food plan and laboratory tests could be stored in the database and after one month, and during hospital visits, the doctor could evaluate the situation of the patient according to the relevancy of the values. Patients can develop productive interactions with their clinicians.

## 5. Conclusions

In conclusion, it is possible for patients and their guardians to incorrectly estimate the amount of amino acids in diets, which might compromise metabolic regulation. Therefore, we created a digital solution to help patients and their caregivers gain better access to dietary information and make keeping track of food intake and planning meals easier. Adherence to medical nutrition treatment and metabolic management should improve because of this sort of personalized medicine due to improved dietary tracking and information sharing between MSUD patients and healthcare providers. In this research MSUD, patients were selected as an example for simplification, but the proposed smart plate and mobile application can be used for other metabolic diseases. For analysis of the productivity of the device, this research will be conducted in the future on two groups of patients: those who have used the device and those who are not. The device software will help to estimate the metabolic disorder type and interact with clinicians. Minerals and vitamins can also be added to patients’ prescriptions to improve their healthcare. Collecting feedback from healthcare providers, patients, and caregivers and incorporating it into the application for future improvement, as well as conducting formal evaluations of user-profiles and behaviors, are also potential next steps. Finally, these technologies are intended solely as a supplementary resource, and should not be used in place of the counsel of a clinician or registered dietitian.

## Figures and Tables

**Table 1:** Summary of most relevant applications that assist IEM patients.

App Name	Developer	Disease	Operating System Availability	Cost	Artificial Intelligence or Computer Vision
Metabolic Diet App [17]	Division of Biochemical Diseases, BC Children’s Hospital, University of British Columbia, Vancouver	16 types of IEMs	Website	Free	No
Dietassistant For MSUD [18]	Julian Waluschyk	MSUD	Android	$3.99	No
Metabolic Balancer [19]	Cambrooke Therapeutics, Inc	PKU	IOS	Free	No
PKU Calculator [20]	Soheb Mahmood	PKU	Windows &Android	$1.99	No
AccuGo for PKU [21]	Jeremy Cross	PKU	IOS	$0.99	No
My Diet for PKU [22]	Healthy Lifestyle	PKU	Android	Free	No
PKU Diet [23]	Viasheslav Gatsko	PKU	Android & IOS	$3.49	No

**Table 2:** The questions and possible answers that were used in the questionnaire.

Questions	Possible Answers
Age group	Less than 12 years 12–18 years Older than 18 years
When did you get diagnosed with this disease?	Since birth Since 6 months More than 6 months
Who is responsible for monitoring your food intake	My parent Myself
In one year، how many times did you change your food plan with your doctor?	Once Twice More than 3 times
Is it hard to monitor your food plan?	Yes No
Would it be helpful for a mobile application to give you a warning about what your consuming?	Yes No
Do you prefer to calculate your food consumption by?	Manual Mobile application
Would you buy a smart device that helps with monitoring your food plan?	Yes No

**Table 3:** Ranges of Adequate Intake for Infants, Children, and Adults With MSUD [1].

Age	Nutrient					
	ILE	LEU	VAL	Protein	Energy	Fluid
	(mg/kg)	(mg/kg)	(mg/kg)	(g/kg)	(kcal/kg)	(mL/kg)
Infants						
0 to <3 mo	36–60	60–100	42–70	3.50–3.00	120 (145–95)	150–125
3 to <6 mo	30–50	50–85	35–60	3.50–3.00	115 (145–95)	160–130
6 to <9 mo	25–40	40–70	28–50	3.00–2.50	110 (135–80)	145–125
9 to <12 mo	18–33	30–55	21–38	3.00–2.50	105 (135–80)	135–120
	(mg/day)	(mg/day)	(mg/day)	(g/day)	(kcal/day)	(mL/day)
Girls and Boys						
1 to <4 y	165–325	275–535	190–400	30.0	1300 (900–1800)	900–1800
4 to <7 y	215–420	360–695	250–490	35.0	1700 (1300–2300)	1300–2300
7 to <11 y	245–470	410–785	285–550	40.0	2400 (1650–3300)	1650–3300
Women						
11 to <15 y	330–445	550–740	385–520	50.0	2200 (1500–3000)	1500–3000
15 to <19 y	330–445	550–740	385–520	55.0	2100 (1200–3000)	1200–3000
19 y	300–450	400–620	420–650	60.0	2100 (1400–2500)	1400–2500
Men						
11 to <15 y	325–435	540–720	375–505	55.0	2700 (2000–3700)	2000–3700
15 to <19 y	425–570	705–945	495–665	65.0	2800 (2100–3900)	2100–3900
19 y	575–700	800–1100	560–800	70.0	2900 (2000–3300)	2000–3300

Reprinted with permission from Ref. [1]. 2001, Acosta, P.B.; Yannicelli, S.

## Data Availability

Publicly available datasets were analyzed in this study. This data can be found here: (https://ieee-dataport.org/open-access/foodd-food-detection-dataset-calorie-measurement-using-food-images (accessed on 1 September 2022).

## Supplementary Materials

The following supporting information can be downloaded at: https://www.mdpi.com/article/10.3390/healthcare11020178/s1, Table S1: Selected Requirements for Laboratories; Table S2: Sample of Comparison of Nutrient Content of High- and Low-Protein Foods at the 100-g Serving Size.

## References

[1] Acosta P.B., Yannicelli S. (2001). The Ross Metabolic Formula System Nutrition Support Protocols.

[2] Blackburn P.R., Gass J.M., Vairo F.P.E., Farnham K.M., Atwal H.K., Macklin S., Klee E.W., Atwal P.S. (2017). Maple syrup urine disease: Mechanisms and management. Appl. Clin. Genet..

[3] Al-Rubeaan K., Bawazeer N., Al Farsi Y., Youssef A.M., Al-Yahya A.A., AlQumaidi H., Al-Malki B.M., Naji K.A., Al-Shehri K., Al Rumaih F.I. (2018). Prevalence of metabolic syndrome in Saudi Arabia—A cross sectional study. BMC Endocr. Disord..

[4] Hazman M., Idrees A.M. A healthy nutrition expert system for children. Proceedings of the 2015 E-Health and Bioengineering Conference (EHB).

[5] Chen C.H., Karvela M., Sohbati M., Shinawatra T., Toumazou C. (2018). PERSON—Personalized Expert Recommendation System for Optimized Nutrition. IEEE Trans. Biomed. Circuits Syst..

[6] Cioara T., Anghel I., Salomie I., Barakat L., Miles S., Reidlinger D., Taweel A., Dobre C., Pop F. (2018). Expert system for nutrition care process of older adults. Futur. Gener. Comput. Syst..

[7] Mikhailava V., Pyshkin E., Klyuev V. (2020). Aesthetic Evaluation of Food Plate Images using Deep Learning. Int. Conf. Adv. Commun. Technol. ICACT.

[8] Palmer S. (1990). Recommended Dietary Allowances.

[9] Abadingo M.E., Abacan M.A.R., Basas J.R.U., Padilla C.D. (2021). Pregnancy in an adolescent with maple syrup urine disease: Case report. Mol. Genet. Metab. Rep..

[10] Tahir G.A., Loo C.K., Moy F.M., Kong N. (2020). Evaluation of Issues, Usability and Functionality of Dietary Related Mobile Applications: A Systematic Literature Review. http://arxiv.org/abs/2008.09883.

[11] Ege T., Yanai K. (2018). Image-based food calorie estimation using recipe information. IEICE Trans. Inf. Syst..

[12] Li J., Guerrero R., Pavlovic V. (2019). Deep cooking: Predicting relative food ingredient amounts from images. MADiMa’19, Proceedings of the 5th International Workshop on Multimedia Assisted Dietary Management.

[13] Lee K.H., He X., Zhang L., Yang L. CleanNet: Transfer Learning for Scalable Image Classifier Training with Label Noise. Proceedings of the IEEE Conference on Computer Vision and Pattern Recognition (CVPR).

[14] He Y., Xu C., Khanna N., Boushey C.J., Delp E.J. (2013). Food Image Analysis: Segmentation, Identification and Weight Estimation. Physiol. Behav..

[15] Mougiakakou S.G., Bartsocas C.S., Bozas E., Chaniotakis N., Iliopoulou D., Kouris I., Pavlopoulos S., Prountzou A., Skevofilakas M., Tsoukalis A. (2010). SMARTDIAB: A Communication and Information Technology Approach for the Intelligent Monitoring, Management and Follow-up of Type 1 Diabetes Patients. IEEE Trans. Inf. Technol. Biomed..

[16] Piniewski B., Muskens J., Estevez L., Carroll R., Cnossen R. (2010). Empowering Healthcare Patients with Smart Technology. Computer.

[17] Hommersom A., Lucas P.J., Velikova M., Dal G., Bastos J., Rodriguez J., Germs M., Schwietert H. MoSHCA—My mobile and smart health care assistant. Proceedings of the 2013 IEEE 15th International Conference on e-Health Networking, Applications and Services (Healthcom 2013).

[18] van Karnebeek C., Ueda K., Cheng B., Giezen A., Ho G., Houben R. (2016). Metabolic Diet App. Division of Biochemical Diseases, BC Children’s Hospital.

[19] Waluschyk J. “Dietassistant For MSUD”, 2021, App Store. https://apps.apple.com/us/app/dietassistant-for-msud/id1512993749.

[20] (2009). Metabolic Balancer. Cambrooke‏ Therapeutics, Inc. https://apps.apple.com/us/app/metabolic-balancer/id335901843.

[21] Mahmood S. (2022). PKU Calculator. https://play.google.com/store/apps/details?id=com.pkucalcapp&hl=en&gl=US.

[22] Cross J. (2020). AccuGo for PKU. https://apptopia.com/ios/app/477075228/intelligence.

[23] (2018). “My Diet for PKU”, Healthy Lifestyle. https://m.apkpure.com/my-diet-for-pku/com.mlashch.mydiet.pku.

[24] Gatsko V. (2021). PKU Diet. http://pkudiet.tilda.ws.

[25] Gardner I. (2016). Chapter 18: Knowledge Acquisition, Representation, and Reasoning. Pearson Educ..

[26] Pouladzadeh P., Yassine A., Shirmohammadi S. (2015). FooDD: Food Detection Dataset for Calorie Measurement Using Food Images. New Trends in Image Analysis and Processing ICIAP 2015 Workshops.

[27] Pouladzadeh P., Shirmohammadi S., Bakirov A., Bulut A., Yassine A. (2015). Cloud-based SVM for food categorization. Multimed. Tools Appl..

[28] Pouladzadeh P., Shirmohammadi S., Yassine A. (2014). Using Graph Cut Segmentation for Food Calorie Measurement. IEEE Int. Symp. Med. Meas. Appl..

[29] Pouladzadeh P., Shirmohammadi S., Almaghrabi A. (2014). Measuring Calorie and Nutrition from Food Image. IEEE Trans. Instrum. Meas..

[30] Saddam Arduino Weight Measurement using Load Cell and HX711 Module. Circuit Digest 2017. https://circuitdigest.com/microcontroller-projects/arduino-weight-measurement-using-load-cell.

[31] Dearborn J.L., Urrutia V.C., Kernan W.N. (2015). The case for diet: A safe and efficacious strategy for secondary stroke prevention. Front. Neurol..

[32] Ho G., Ueda K., Houben R.F., Joa J., Giezen A., Cheng B., van Karnebeek C.D. (2016). Metabolic Diet App Suite for inborn errors of amino acid metabolism. Mol. Genet. Metab..

