# Supplementary material for: A Smart Monitoring System for Self-Nutrition Management in Pediatric Patients with Inherited Metabolic Disorders: Maple Syrup Urine Disease (MSUD)
Source: Healthcare (Basel). 2023 Jan 6;11(2):178. doi: 10.3390/healthcare11020178 (PMC9859191; doi:10.3390/healthcare11020178)
Supplement: Supplementary file 1 [file healthcare-11-00178-s001.zip › healthcare-2130654-supplementary.pdf]

Table S1 Selected Requirements for Laboratories [1]

Parameter	Age of Patient		Status [1]	
		Normal	Marginal	Deficient
**Albumin, plasma (g/dL)**				
Infants and children	< 1 yr	4.00 - 5.00	< 3.50	3.00
	1 to < 9 yr	3.50 - 5.00	< 3.50	3.00
	≥ 9 yr	3.50 - 5.00	< 3.50	3.00
Pregnant women	Trimester 1	≥ 4.00	≤ 3.50	≤ 3.00
	Trimester 2	≥ 3.75	≤ 3.25	≤ 2.75
	Trimester 3	≥ 3.50	≤ 3.00	≤ 2.50
**Transthyretin (mg/dL)**	All ages and during pregnancy	22	< 20	< 15
**B_{12}, serum, (pg/mL)**	All ages and during pregnancy	> 300	≤ 260	≤ 200
**Cholesterol (mg/dL)**				
Infants, children, youngadults	0 < 20 yr	150 - 200	< 150	< 120
Pregnant women	Trimester 1	150 - 250	< 150	< 120
	Trimester 2	150 - 350	< 150	< 120
	Trimester 3	200 - 400	< 200	< 150
**Ferritin, plasma (ng/mL)**				
Infants and children	6 mo to < 2 yr	≥ 30	≤ 20	≤ 12
	2 to < 6 yr	≥ 40	≤ 20	≤ 12
	6 to < 12 yr	≥ 50	≤ 20	≤ 12
	≥ 12 yr	≥ 50	≤ 20	≤ 12
Pregnant women	Trimester 1	≥ 50	≤ 20	≤ 12
	Trimester 2	≥ 40	≤ 20	≤ 12
	Trimester 3	≥ 30	≤ 20	≤ 12
**Folate, erythrocyte (ng/mL)**	All ages and during pregnancy	≥ 200 < 300	≤ 160	≤ 120
**Hemoglobin (Hgb) (g/dL)**				
Infants and children	0 to < 5 yr	≥ 11.0	< 11.0	< 10.0
	5 to < 8 yr	≥ 11.5	< 11.5	< 10.5
	8 to < 12 yr	≥ 12.0	< 12.0	< 11.0
Girls and women	≥ 12 yr	≥ 12.0	< 12.0	< 11.0
Pregnant women	Trimester 1	≥ 12.0	< 12.0	< 11.0
	Trimester 2	≥ 12.0	< 12.0	< 11.0
	Trimester 3	≥ 12.0	< 12.0	< 11.0
Boys and men	12 to < 15 yr	≥ 12.5	< 12.5	< 11.5
	15 to < 18 yr	≥ 13.0	< 13.0	< 12.0
	≥18 yr	≥ 14.0	< 14.0	< 13.0
**Hematocrit (Hct) (%)**				
Infants and children	0 to < 2 yr	≥ 33	< 33	< 30
	2 to < 5 yr	≥ 34	< 34	< 32
	5 to < 8 yr	≥ 35	< 35	< 33
	8 to < 12 yr	≥ 36	< 36	< 33
Girls and women	12 to < 18 yr	≥ 36	< 36	< 33
	≥18 yr	≥ 37	< 37	< 33
Pregnant women	Trimester 1	≥ 36	< 36	< 33
	Trimester 2	≥ 36	< 36	< 33
	Trimester 3	≥ 36	< 36	< 33
Boys and men	12 to < 15 yr	≥ 37	< 37	< 34
	15 to < 18 yr	≥ 38	< 38	< 36
	> 18 yr	> 40	< 40	< 39

Table S2 Sample of Comparison of Nutrient Content of High- and Low-Protein Foods at the 100-g Serving Size [1]

Food	Protein	Fat	Energy	ARG	CYS	HIS	ILE	LEU	LYS	MET	PHE	THR	TRP	TYR	VAL
	(g)	(g)	(kcal)	(mg)	(mg)	(mg)	(mg)	(mg)	(mg)	(mg)	(mg)	(mg)	(mg)	(mg)	(mg)
**A protein ® Foods**															
Cracker toast	1.7	4.4	398	100	20	40	50	10	80	30	60	60	20	30	70
dp Baking mix, low protein	0.3	9.4	410	20	5	6	4	8	8	1	5	3	3	3	10
dp Chocolate chip cookies	0.8	22.9	497	30	10	10	70	10	20	2	10	20	10	3	10
Pasta, uncooked	0.5	0.9	360	30	9	10	10	50	20	10	20	10	5	10	20
Rusks	0.8	7.2	420	30	10	10	30	50	40	10	30	30	10	20	30
Wheat Starch	0.5	0.1	350	30	9	10	10	50	20	10	20	10	5	10	20
**Dietary Specialties**															

**Breads**															
bread machine mix	0.3	6.0	351	20	5	7	10	20	10	10	10	10	3	10	10
dinner rolls	0.5	3.5	246	30	9	10	20	40	40	10	20	20	7	10	20
rye-wheat bread	0.8	3.0	200	40	10	10	30	50	40	10	30	30	9	20	40
wheat starch bread	0.4	4.4	242	10	8	9	10	20	9	7	10	10	5	10	20
**Cheese**															
imitation	2.2	20.0	267	80	30	50	120	220	160	50	90	120	20	80	80
sauce mix powder	6.0	26.0	482	170	110	110	190	320	460	90	150	370	90	120	260
slices	3.5	26.3	316	120	30	90	170	270	290	80	150	180	40	160	190
Chocolate bon ons	2.2	11.5	436	100	20	50	100	180	130	40	100	90	30	90	140
Chocolate sauce mix	0.2	3.0	375	10	4	3	4	6	6	2	4	4	3	2	5
**Cookies**															
butterscotch	0.6	22.9	500	20	8	10	30	60	40	10	30	20	9	20	40
chocolate tea	0.7	19.0	482	30	10	10	20	60	20	10	30	20	7	20	30
coconut	0.8	23.0	503	60	10	20	30	90	20	10	40	20	4	20	40
ginger	0.3	11.0	412	10	6	8	10	30	6	6	10	9	1	10	10
orange	0.3	20.0	489	10	7	9	10	40	7	7	10	10	1	10	10
**Fruit products**															
cassava chips	1.7	26.7	520	160	30	20	30	40	50	10	30	30	20	20	40
plantain chips	2.4	38.0	571	190	30	110	60	100	110	30	80	60	20	50	80
Graham cracker	0.5	20.3	473	30	9	10	10	30	20	10	20	10	5	10	20
**Grains, uncooked**															
Porridge	0.3	1.0	360	10	6	8	9	20	8	3	10	8	2	10	10
Rice, imitation	0.3	1.0	360	10	6	8	9	20	8	3	10	8	2	10	10
Pasta, uncooked	0.3	1.0	360	10	6	8	8	20	8	3	10	8	2	10	10
Tomato sauce	2.8	2.0	360	270	20	40	40	80	110	20	70	60	20	30	80
**Breads**															
mix	0.3	0.1	376	8	7	4	6	10	9	3	6	6	3	2	9
rice	0.3	8.7	325	10	5	7	5	10	10	6	10	10	3	6	10
rice starch	0.4	9.5	334	10	7	8	10	30	20	4	10	10	4	4	20
wheat starch	0.5	5.0	257	10	8	8	10	20	20	4	10	10	4	9	10
Cheese, cheddar or mozzarella	1.9	20.0	279	100	10	80	140	280	220	60	150	110	30	120	180
**Cookies**															
lemon sandwich	0.2	21.0	486	10	4	3	4	6	6	2	4	4	3	2	5
lemon shortbread	0.2	21.5	476	10	2	2	6	10	6	3	8	5	2	7	8
orange shortbread	0.4	19.2	469	10	10	5	10	10	5	3	10	10	10	5	10
Egg replacer	0.1	0.0	375	4	1	1	1	3	3	1	2	1	1	1	3
Flour															
potato starch	0.1	0.0	320	7	1	2	2	4	5	1	4	2	1	2	4
rice starch	0.8	0.0	375	40	7	8	20	40	20	10	30	10	6	20	30
tapioca	0.1	0.0	396	10	2	2	2	3	3	1	2	2	2	1	3
**Gel mix**															
banana	0.0	0.0	372	0	0	0	0	0	0	0	0	0	0	0	0
chocolate	0.0	0.0	344	0	0	0	0	0	0	0	0	0	0	0	0
lemon	0.0	0.0	367	0	0	0	0	0	0	0	0	0	0	0	0
lime	0.0	0.0	383	0	0	0	0	0	0	0	0	0	0	0	0
orange	0.0	0.0	361	0	0	0	0	0	0	0	0	0	0	0	0
raspberry	0.0	0.0	370	0	0	0	0	0	0	0	0	0	0	0	0
strawberry	0.0	0.0	372	0	0	0	0	0	0	0	0	0	0	0	0
Ice cream cone	0.1	2.9	396	10	2	2	2	3	3	1	2	2	2	2	3
Pizza shells	1.7	13.6	320	3	7	10	10	20	20	9	10	10	4	5	20
**Potato**															
chips	0.7	49.0	641	40	10	10	20	40	20	6	20	10	10	10	20
mix	0.1	0.0	380	7	1	2	2	4	5	1	3	2	1	2	4
Pumpkin donut	0.8	14.0	304	40	5	10	20	30	30	10	20	20	9	30	30
Waffles	0.2	7.0	248	20	1	8	10	30	10	8	20	10	1	10	20
Cake base	0.3	8.8	353	10	6	6	10	6	5	5	10	7	3	8	10
**Cookies**															
chocolate chip	0.8	20.0	500	30	10	10	20	50	10	10	30	20	9	20	30

References

[1] P. B. Acosta and S. Yannicelli, *The Ross Metabolic Formula System Nutrition Support Protocols*, 4th editio. Ross Products Division/, 2001.
